# Global Proteomic Analysis of Lysine Crotonylation in the Plant Pathogen *Botrytis cinerea*

**DOI:** 10.3389/fmicb.2020.564350

**Published:** 2020-10-23

**Authors:** Ning Zhang, Zhenzhou Yang, Wenxing Liang, Mengjie Liu

**Affiliations:** Key Lab of Integrated Crop Pest Management of Shandong Province, College of Plant Health and Medicine, Qingdao Agricultural University, Qingdao, China

**Keywords:** crotonylome, *Botrytis cinerea*, LC-MS/MS, fungal pathogenicity, lysine crotonylation

## Abstract

Lysine crotonylation (Kcr), a recently discovered post-translational modification, plays a key role in the regulation of diverse cellular processes. *Botrytis cinerea* is a destructive necrotrophic fungal pathogen distributed worldwide with broad ranging hosts. However, the functions of Kcr are unknown in *B. cinerea* or any other plant fungal pathogens. Here, we comprehensively evaluated the crotonylation proteome of *B. cinerea* and identified 3967 Kcr sites in 1041 proteins, which contained 9 types of modification motifs. Our results show that although the crotonylation was largely conserved, different organisms contained distinct crotonylated proteins with unique functions. Bioinformatics analysis demonstrated that the majority of crotonylated proteins were distributed in cytoplasm (35%), mitochondria (26%), and nucleus (22%). The identified proteins were found to be involved in various metabolic and cellular processes, such as cytoplasmic translation and structural constituent of ribosome. Particularly, 26 crotonylated proteins participated in the pathogenicity of *B. cinerea*, suggesting a significant role for Kcr in this process. Protein interaction network analysis demonstrated that many protein interactions are regulated by crotonylation. Furthermore, our results show that different nutritional conditions had a significant influence on the Kcr levels of *B. cinerea*. These data represent the first report of the crotonylome of *B. cinerea* and provide a good foundation for further explorations of the role of Kcr in plant fungal pathogens.

## Introduction

Post-translational modifications (PTMs), in which functional groups are covalently introduced to amino acid residues, play important roles in regulating diverse biological processes (Khoury et al., 2011). With the application of liquid chromatography-mass spectrometry (LC-MS/MS)-based proteomics, an array of novel PTMs have been identified. Particularly, lysine residues are targeted by numerous PTMs, including acetylation, succinylation, butyrylation, propionylation, 2-hydroxyisobutyrylation, glutarylation, malonylation, lactylation, and crotonylation (Chen et al., 2007; Tan et al., 2011; Dai et al., 2014; Hirschey and Zhao, 2015; Zhang et al., 2019). Currently, there is a lack of research on the identification of substrates of these PTMs and elucidation of their functional impacts.

Lysine crotonylation (Kcr) was first identified on histones and its role in regulation of gene transcription has been well investigated (Tan et al., 2011). HDAC1, a member of the class I histone deacetylases (HDACs), was found to catalyze histone decrotonylation in mammalian cells (Wei et al., 2017a). Therefore, it is presumed that both protein crotonylation and acetylation are regulated by histone acetyltransferases (HATs) and HDACs. Other than histone proteins, crotonylation is newly discovered targeting non-histone proteins, regulating protein stability, enzymatic activity, protein localization, and many other cellular processes (Wei et al., 2017b; Xu et al., 2017). Advancements in novel enrichment strategies (LC-MS/MS) have made it possible to investigate Kcr on a proteomic level and large number of crotonylated proteins have been identified. Up to now, several organisms or cell lines have been reported with respect to Kcr, including *Carica papaya* (Liu K. et al., 2018), *Nicotiana tabacum* (Sun et al., 2017), *Oryza sativa* (Liu S. et al., 2018), *Danio rerio* (Kwon et al., 2018), and four human cell lines (Wei et al., 2017b; Wu et al., 2017; Xu et al., 2017; Huang et al., 2018). However, the understanding of the features of Kcr is still insufficient. Particularly in plant fungi, global identification of this kind of PTM has not been well reported.

*Botrytis cinerea* is an worldwide economically important fungal pathogen that can cause gray mold disease on a wide variety of hosts (Williamson et al., 2007). In recent years, extensive research has been conducted to interpret transcriptional regulation of virulence genes in *B. cinerea* (Weiberg et al., 2013; Brandhoff et al., 2017; Wang et al., 2017, 2018; Porquier et al., 2019). However, gaps in the protein level studies limited a deeper understanding of the molecular basis of *B. cinerea* pathogenesis. In this study, we conducted the first proteome-wide Kcr analysis in *B. cinerea*. In total, 3967 Kcr sites in 1041 proteins were identified. The crotonylated proteins were distributed in multiple compartments and associated with diversified biological processes. Particularly, 26 crotonylated proteins were found to be associated with virulence of *B. cinerea*. These proteins participates in diverse pathogenesis pathways including signal transduction, redox homeostasis, plant cell wall degrading, secretory of virulence factors, and secondary metabolites biosynthesis. This work provides an extensive dataset for further investigating the physiological role of Kcr in *B. cinerea* and other filamentous fungal pathogens.

## Materials and Methods

### Protein Extraction From *B. cinerea*

The *B. cinerea* strain B05.10 was cultured on PDA plates (2% dextrose, 20% potato, and 1.5% agar) at 25°C for 10 days, and then the conidia were collected and transferred into YEPD medium (2% glucose, 1% yeast extract, and 2% peptone) with shaking at 180 rpm for 14 h. The protein extraction was performed as previously described (Zhou et al., 2016). Briefly, the harvested mycelia were ground into powder in liquid nitrogen. Then the powder sample was suspended by lysis buffer (50 mM nicotinamide, 8 M urea, 65 mM dithiothreitol, 1% Triton-100, 0.1% protease inhibitor cocktail, 2 mM EDTA, and 3 μM Trichostatin A) and then sonicated. After centrifugation at 15,000 × *g* at 4°C for 15 min, 15% cold TCA was used to precipitate the proteins at −20°C for 2 h. The precipitates were washed three times with cold acetone after centrifugation at 4°C for 15 min. Finally, the target protein was re-dissolved in 8 M urea supplemented with 100 mM NH_4_CO_3_ (pH 8.0) and 2-D Quant kit (GE Healthcare) was used to determine protein concentration according to the manufacturer’s instructions.

### Affinity Enrichment of Lysine Crotonylated Peptides

For affinity enrichment, the *B. cinerea* proteins were firstly digested into peptides by trypsin. The digestion reaction was performed with a previously described experimental approach (Zhou et al., 2016), briefly, the trypsin was added at 1:50 trypsin-to-protein mass ratio at the first time overnight and added again at 1:100 trypsin-to-protein mass ratio for another 4 h. The sample was separated into fractions by high PH reverse-phase HPLC using Agilent 300 Extend C18 column (5 μM particles, 4.6 mm ID, and 250 mm length) (Liu L. et al., 2016). The peptides were separated firstly into 80 fractions with a gradient of 2 to 60% acetonitrile in 10 mM (NH_4_)_2_CO_3_ (PH 10.0). Then, the peptides were combined into 8 fractions and dried by vacuum centrifuging. For Kcr peptides enrichment, the tryptic peptides were dissolved in NETN buffer (1 mM EDTA, 100 mM NaCl, 0.5% NP-40, and 50 mM Tris–HCl pH 8.0) and then separated into several fractions. Each fraction was incubated with pan anti-Kcr antibody (PTM-502, PTM Biolabs) conjugated agarose beads overnight at 4°C with gentle shaking. Then the peptides bound with beads were eluted with 0.1% trifluoroacetic acid after washing with NETN buffer and then the acquired peptides were cleaned with C18 Zip Tips (Millipore) (Sun et al., 2019).

### LC-MS/MS Analysis

Liquid chromatography-mass spectrometry analysis of the crotonylated peptides was performed as described (Li et al., 2016; Liu L. et al., 2016; Lv et al., 2016; Zhou et al., 2016) by Micrometer Biotech Company (Hangzhou, China). Briefly, The Kcr peptides were separated using a reversed-phase analytical column (Acclaim PepMap RSLC C18 column, Thermo Scientific) on UPLC system. The gradient was composed of an increase from 2% formic acid (0.1%) to 10% formic acid (0.1% in 98% acetonitrile) for 6 min, 10 to 20% for 45 min, 20% climbing to 80% in 7 min and then holding at 80% at least for 4 min, all maintaining a flow rate of 250 nl/min. The peptides were subjected by to ESI/NSI sources followed by MS/MS in Q Exactive^TM^ Plus (Thermo Scientific) coupled online to UPLC. The Orbitrap was used to detect whole peptides and ion fragments at a resolution of 70,000 and 17,500, respectively, with NCE set at 30. The electrospray voltage was set at 2.0 kV. Automatic gain control (AGC) was used to avoid ion trap overfilling. The m/z range was from 350 to 1800 for MS scans. The MS fixed first mass was set at 100 m/z.

### Generation of Bcpck1-GFP Strains

To generate Bcpck1-GFP overexpression construct, CDS (coding domain sequence) of *Bcpck1* was cloned into pOPT-GFP vector that contains *oliC* promoter, *niaD* terminator, hygromycin phosphotransferase and optimized C-terminal eGFP sequence (Leroch et al., 2011). The constructed plasmid was then transformed into the B05.10 strain using protoplast transformation of *B. cinerea* (Gronover et al., 2001). The resulting transformants were selected by 100 μg/ml hygromycin B.

### Immunoprecipitation and Western Blot Analysis

Total proteins were extracted using lysis buffer (0.5 mM EDTA, 150 mM NaCl, 0.5% NP-40, and 10 mM Tris–HCl pH 7.5) from mycelium of *B. cinerea* cultivated in liquid YEPD or MM (10 mM K_2_HPO_4_, 10 mM KH _2_PO_4_, 4 mM (NH_4_)_2_SO_4_, 2.5 mM NaCl, 2 mM MgSO_4_, 0.45 mM CaCl_2_, 9 mM FeSO_4_, 50 mM glucose, and 1 L water, pH 6.5) with shaking at 180 rpm for 14 h. Anti-GFP agarose beads (KTSM1301, KT HEALTH) were subsequently added and the mixture was incubated for 2 h. The beads were washed 3 times with 500 μl of lysis buffer, and the bound proteins were eluted with SDS-PAGE loading buffer.

The obtained proteins were then separated by 12% SDS-PAGE and immunoblotted using anti-GFP antibody (ab290, Abcam) and anti-Kcr antibody, respectively. Proteins were visualized using Immobilon Western Chemiluminescent HRP Substrate (Millipore) according to the manufacturer’s protocol.

### Database Search

MaxQuant and Andromeda search engine (v.1.5.1.8) were used to analyze the raw data of MS/MS (Cox and Mann, 2008; Cox et al., 2009). The tandem mass spectra collected were searched against *B. cinerea* B05.10 database from UniProt. Mass errors of fragment ions and precursor were set as 0.02 Da and 10 ppm, respectively. Trypsin/P was specified as cleavage enzyme allowing up to 4 missing cleavage, 5 charges and 5 modifications per peptide. Carbamidomethylation on Cysteine was specified as fixed modification and crotonylation on lysine was fixed as variable modification. The minimal peptide was set to seven, and the false discovery rate (FDR) threshold for modification sites and peptides were set as 1%. The Kcr site localization probability of <0.75 was exclude (Zhou et al., 2016; Yang et al., 2018).

### Bioinformatics Analysis

Gene ontology (GO) of crotonylation proteome was performed from the UniProt-GOA database based on three categories: cellular component, molecular function, and biological process according to Zhou et al. (2016). The soft WoLF PSORT was used to predict the subcellular localization of the crotonylated protein (Horton et al., 2007). Proteins secondary structures (β-strand, α-helix, coil) were analyzed by the online tool NetSurfP (Chou and Schwartz, 2011). Soft MoMo (motif-x algorithm) was used to analyze the sequences model of crotonylated proteins composed of amino acids in distinct positions of modify-21-mers (10 amino acids up- and downstream of the Kcr site) in all protein sequences. To define the conservation of crotonylation, the BLASTP was used to compare the crotonylated protein sequences of *B. cinerea* with *Carica papaya* (Liu K. et al., 2018), *Nicotiana tabacum* (Sun et al., 2017), *Oryza sativa* (Liu S. et al., 2018), *Danio rerio* (Kwon et al., 2018), and four human cell lines including *Human peripheral blood*, *Human Hela cells*, *Human H1299 cells*, and *Human A549 cells* (Wei et al., 2017b; Wu et al., 2017; Xu et al., 2017; Huang et al., 2018). InterProScan was used to perform the protein domain functional annotation based on protein alignment method and the InterPro domain database. Kyoto Encyclopedia of Genes and Genomes (KEGG) database was employed to annotate protein pathway description (Kanehisa et al., 2004). Cytoscape software was used to analyze the protein-protein interactions which was obtained from the STRING database (Shannon et al., 2003; Szklarczyk et al., 2015). A two-tailed Fisher’s exact test was used to verify the enrichment of lysine crotonylated proteins against all database proteins. All projects with a corrected *p*-valve < 0.05 is considered significant.

## Results

### Identification and Analysis of Lysine-Crotonylated Sites and Proteins in *B. cinerea*

Lysine crotonylation is a newly discovered PTM which is poorly studied in *B. cinerea*, one of the most important plant fungi pathogen in the world. To extensively characterize protein crotonylation in *B. cinerea*, a proteome-wide analysis of Kcr was carried out. Proteins extracted from mycelium were digested by trypsin and the crotonylation peptides were enriched using anti-crotonyl lysine antibody, followed by high-resolution LC-MS/MS analysis. An overview of the experimental procedures was demonstrated in Supplementary Figure S1A. Mass errors of the identified peptides were checked first to confirm the dependability of the MS data. As shown in Supplementary Figure S1B, the distribution of mass errors was near zero, most were less than 10 PPM, indicating that the mass accuracy fits the requirement. Moreover, the length of most lysine-crotonylated peptides were between 7 and 20, which is consistent with tryptic peptides (Supplementary Figure S1C). The above results demonstrated that the sample preparation meets the standard.

After a global lysine crotonylome analysis using LC-MS/MS, 4913, 4839, and 4922 Kcr sites in 1242, 1230, and 1253 crotonylated proteins, respectively, were identified within three independent biological repeats. Moreover, 3967 Kcr sites and 1041 crotonylated proteins were overlapped in the individual triplicate experiments (Supplementary Figure S2 and Supplementary Table S1). MS/MS spectra of three crotonylated peptides were shown in Supplementary Figure S3. The number of crotonylated sites in the identified proteins was then calculated. As shown in Supplementary Figure S4, 371 (35%) proteins contained only one crotonylation site, whereas 670 (65%) proteins had multiple crotonylation sites.

### Pattern Analysis of Crotonylated Sites

**FIGURE 1:**
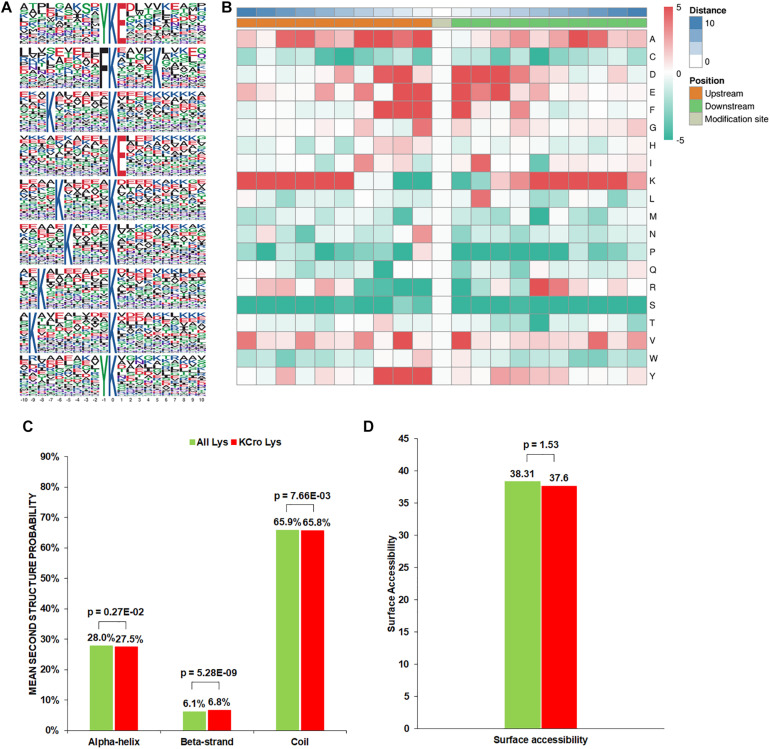
Properties of the Kcr peptides in *B. cinerea*. **(A)** Crotonylation sequence motifs for ±10 amino acids surrounding the Kcr sites. **(B)** Heatmap of the amino acid compositions of the Kcr sites demonstrating the frequency of certain amino acids around the modified lysine. Red indicates high frequency and green means low frequency. **(C)** Probabilities of Kcr in the structures of beta-strand, alpha-helix, and coil. **(D)** Predicted surface accessibility of Kcr sites.

To evaluate the properties of Kcr sites in *B. cinerea*, we examined the sequence motif flanking the identified peptides. A total of nine conserved amino acid sequences from −10 to +10 around the crotonylated lysine were extracted from 3196 peptides (Figure 1A). Particularly, motifs YKcrE, FKcr, and KcrE were strikingly conserved. Among them, FKcr and KcrE have been identified as crotonylation motifs previously, while YKcrE was firstly found in our study, which may represent a characteristic feature of crotonylation in *B. cinerea*. To further analyze these motifs, heatmaps of the amino acid sequences surrounding the crotonylation sites were generated. The results showed that certain amino acid residues surrounding the Kcr were markedly enriched. K residues were observed to be enriched in the −10 to −5 and +5 to +9 positions, while residues A, D, E, F, Y were significantly enriched in −4 to −1, −3 to +3, −2 to +3, −3 to +1, and −3 to −1 positions, respectively (Figure 1B).

In order to clarify the relationship between crotonylation and the presence of protein structures in *B. cinerea*, a structure analysis of the crotonylated proteins was performed. As shown in Figure 1C, 34.3% of the crotonylated sites were located in regions with ordered secondary structures. Among them, 27.5% were located in alpha-helix, and 6.8% were in a beta-strands. The residual 65.8% of the crotonylated sites were distributed in disordered protein regions. However, considering the distribution patterns of crotonylated lysines and all lysines are similar, there was no tendency of Kcr in *B. cinerea*. The surface accessibility of Kcr sites was further evaluated. The results showed that the exposure of crotonylation sites on the protein surface is close to that of all lysine residues (Figure 1D). Therefore, Kcr may not be affected by the surface properties of proteins in *B. cinerea*.

### Conservation Analysis of Lysine Crotonylated Proteins

**FIGURE 2:**
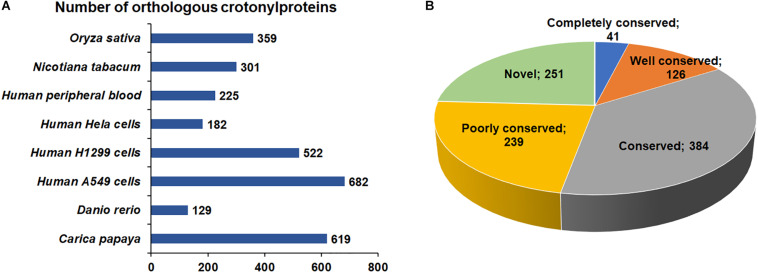
Conservation analysis of lysine crotonylated proteins. **(A)** Number of orthologous crotonylproteins in eight organisms or cell lines with reported crotonylomes. **(B)** A pie chart of conservation of crotonylproteins in *Oryza sativa*, *Nicotiana tabacum*, *Human peripheral blood*, *Human Hela cells*, *Human H1299 cells*, *Human A549 cells*, *Danio rerio*, and *Carica papaya*. Classification was performed as follows: Completely conserved group, 8 orthologs; Well conserved group, 6 to 7 orthologs; Conserved group, 3 to 5 orthologs; Poorly conserved group, 1 to 2 orthologs; Novel group, 0 orthologs.

Increasing studies in recent years have revealed improved number of crotonylated proteins in various species or cell lines (Sun et al., 2017; Wei et al., 2017b; Wu et al., 2017; Xu et al., 2017; Huang et al., 2018; Kwon et al., 2018; Liu K. et al., 2018; Liu S. et al., 2018). However, the conservation of Kcr in these organisms is unknown. As such, we compared the crotonylated proteins in *B. cinerea* with those in eight organisms or cell lines that have determined crotonylomes. Totally, 3019 orthologs of the crotonylproteins in *B. cinerea* were identified in these organisms (Supplementary Table S2). As shown in Figure 2A, 790 crotonylated proteins have orthologs in *Oryza sativa* (359 proteins), *Nicotiana tabacum* (301 proteins), *Human peripheral blood* (225 proteins), *Human Hela cells* (182 proteins), *Human H1299 cells* (522 proteins), *Human A549 cells* (682 proteins), *Danio rerio* (129 proteins), and *Carica papaya* (619 proteins), which account for 75.9% (790/1041 proteins) of the total crotonylproteins in *B. cinerea*. We further classified the crotonylated proteins of *B. cinerea* depending on the number of the orthologous proteins in these organisms. The data demonstrated that the percentage of completely conserved proteins (have 8 orthologs), well-conserved proteins (have 6 to 7 orthologs), conserved proteins (have 3 to 5 orthologs) and poorly conserved proteins (have 1 to 2 orthologs) were 3.9% (41/1041 proteins), 12.1% (126/1041 proteins), 36.9% (384/1041 proteins), and 22.9% (239/1041 proteins) (Figure 2B), respectively. Furthermore, 24.1% (251/1041 proteins) of the crotonylated proteins in *B. cinerea* was characterized as novel proteins since no orthologs were found in these organisms (Figure 2B). These results suggest that Kcr plays both common and specific roles in different species.

### Functional Annotation and Cellular Localization of Crotonylated Proteins in *B. cinerea*

**FIGURE 3:**
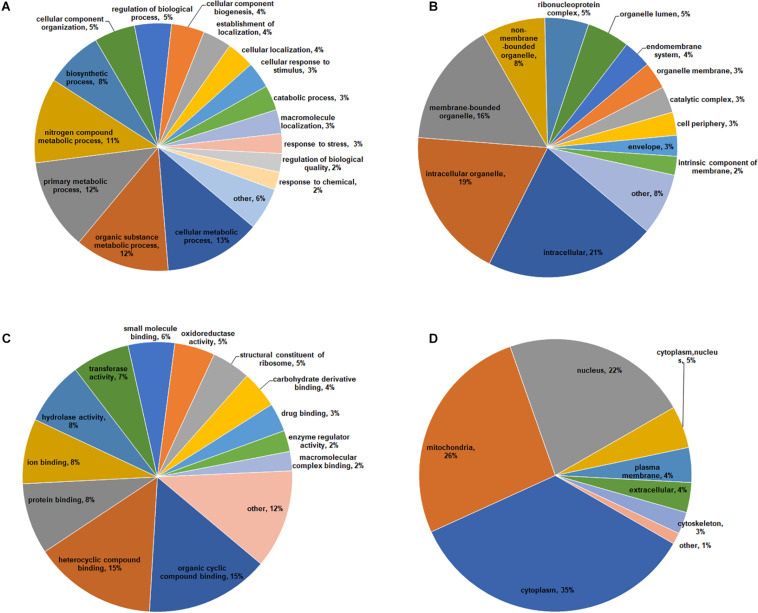
Functional classification of lysine crotonylated proteins based on GO and subcellular location in *B. cinerea*. GO classification of crotonylated proteins based on biological process **(A)**, cellular component **(B)**, and molecular function **(C)**. **(D)** Subcellular localization classification of the crotonylated proteins.

To obtain a comprehensive overview of potential roles of crotonylated proteins in *B. cinerea*, we conducted the Gene Ontology (GO) functional classification analysis of all crotonylated proteins based on their biological process, cellular component, and molecular function (Figure 3 and Supplementary Table S3). The result of biological process analysis showed that crotonylated proteins were widely distributed within different kinds of metabolic and cellular processes (Figure 3A). For cellular component analysis, crotonylated proteins were distributed among various cellular compartments, mostly in intracellular (21%), intracellular organelle (19%), and membrane-bounded organelle (16%) (Figure 3B). Within the molecular function category, the majority of the modified proteins were composed of those related to heterocyclic compound binding (15%), organic cyclic compound binding (15%), protein binding (8%), ion binding (8%), and hydrolase activity (8%) (Figure 3C).

Subcellular localization analysis of the crotonylated proteins was also performed. As shown in Figure 3D, a large number of the crotonylated proteins in *B. cinerea* were distributed in the cytoplasm (35%), mitochondria (26%), and nucleus (22%). These data demonstrated that the crotonylated proteins, with diversified cellular distribution, are involved in various biological processes in *B. cinerea*.

### Functional Enrichment Analysis of Crotonylated Proteins

**FIGURE 4:**
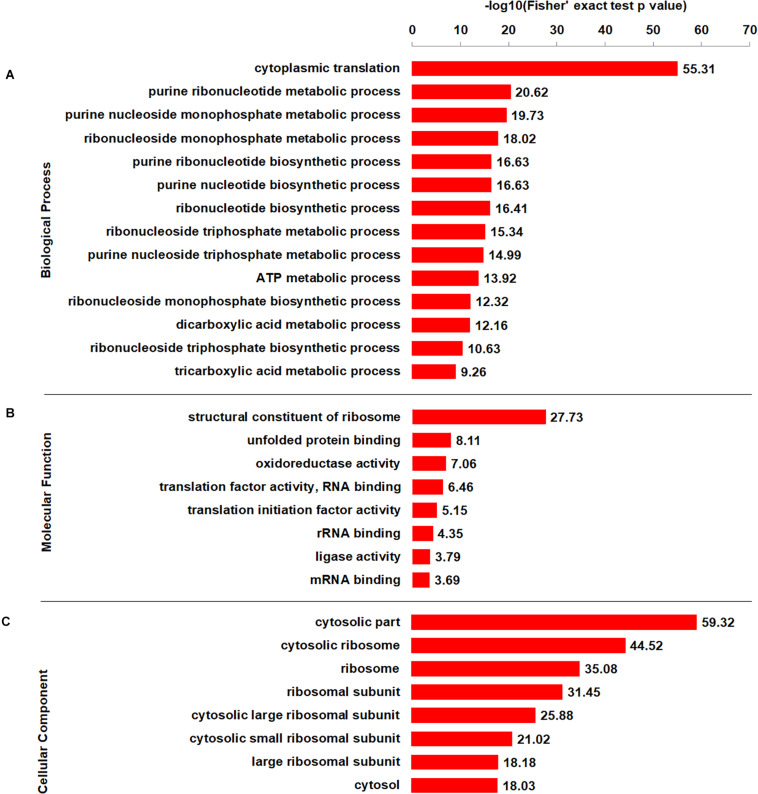
GO-based enrichment analysis of crotonylated proteins according to biological process **(A)**, molecular function **(B)**, and cellular component **(C)**.

To better elucidate the characteristics of crotonylaed proteins in *B. cinerea*, enrichment analyses of GO (Gene Ontology), protein domain and KEGG pathway were conducted. The enrichment analysis of biological process revealed that the majority of crotonylaed proteins were related to cytoplasmic translation (Figure 4A and Supplementary Table S4). In support of these observations, large numbers of the modified proteins were involved in structural constituent of ribosome in molecular function enrichment analysis (Figure 4B and Supplementary Table S4). Based on cellular component enrichment analysis, proteins located to cytosolic part were more likely to be crotonylated (Figure 4C and Supplementary Table S4).

Further enrichment analyses of protein domain and KEGG pathway obtained similar results. The protein domain enrichment analysis demonstrated that the proteins with domains of proteasome subunit were more likely to have crotonylation (Supplementary Figure S5A and Supplementary Table S5). Consistent with these findings, enrichment analysis of KEGG pathway indicated that a large proportion of crotonylated proteins were associated with ribosome (Supplementary Figure S5B and Supplementary Table S6). Overall, our data indicate that lysine crotonylated proteins are widely distributed and involved in various pathways, suggesting important roles of crotonylation in cell metabolism.

### Analysis of Crotonylated Proteins Related to Fungal Pathogenicity

**FIGURE 5:**
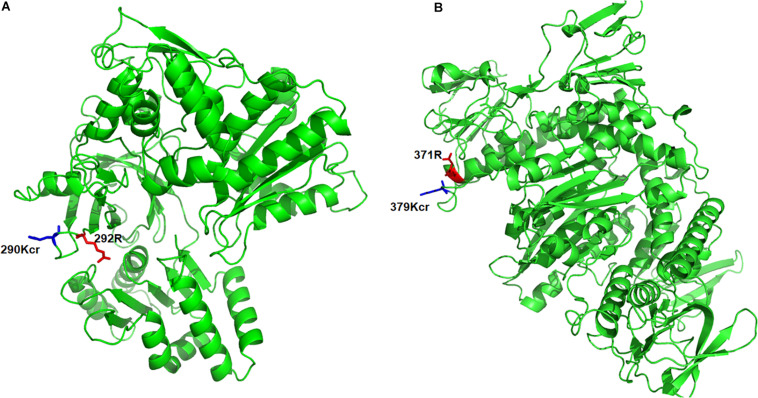
Three-dimensional structure of Bccpr1 **(A)** and Bcglr1 **(B)** with identified crotonylation site. The structure was derived from PDB database. The crotonylated lysine residues and adjacent arginine residues were indicated by blue and red colored sticks, respectively.

To further understand the pathogenesis regulated by crotonylation in *B. cinerea*, we summarized the crotonylated proteins reported to be involved in fungal pathogenicity and listed in Table 1 and Supplementary Table S7. A total of 26 proteins were identified, with diversified numbers of Kcr sites varied from one to eight. These proteins participate in diverse pathogenesis pathways including signal transduction (6 proteins), redox homeostasis (6 proteins), plant cell wall degrading (4 proteins), secretory of virulence factors (3 proteins), and secondary metabolites biosynthesis (7 proteins), indicating that Kcr has a broad range of effects on pathogenicity of *B. cinerea*. Importantly, most of the crotonylated sites were located or near to the functional domains of these proteins. For example, cytochrome P450 reductase (Bccpr1) was found to be crotonylated at K290, which is next to R292, one of the conserved key sites of flavin adenine dinucleotide (FAD) domain of Cytochrome P450 reductase (Ebrecht et al., 2019; Figure 5A). Glutathione reductase (Bcglr1) carried the crotonylated K379 which is spatially close to one of the key sites of this enzyme, R371 (Yu and Zhou, 2007; Figure 5B). These data suggests that Kcr may have effect on the adjacent key domain sites, and thus regulate the function of these proteins involved in pathogenicity.

**TABLE 1:** Crotonylated proteins associated with pathogenicity in *B. cinerea*.

Protein name	Protein description	Functional Classification
Bclga1	Dihydrodipicolinate synthase	
Bcgar1	Aldo/keto reductase	Plant cell wall degrading
Bclgd1	Galactonate dehydratase	
Bcpg1	Glycosyl hydrolase	
Bctrx1	Thioredoxin	
Bctrx2	Thioredoxin	
Bctrr1	Thioredoxin reductase	
Bcglr1	Glutathione reductase	Redox homeostasis
Bcpdi1	Protein disulfide-isomerase	
Bcsod1	Superoxide dismutase [Cu-Zn]	
Bmp1	Mitogen-activated protein kinase	
Bmp3	Mitogen-activated protein kinase	
Bcptc3	Serine/threonine protein phosphatase	Signal transduction
Bcras1	Ras-related GTPase	
Bcpka1	cAMP-dependent protein kinase	
Bcg1	G-protein alpha subunit	
Bcbrn1	Tetrahydroxynaphthalene Reductases	
Bcbrn2	Tetrahydroxynaphthalene Reductases	
Bcscd1	Scytalone dehydratase	
Bcp1	Peptidyl-prolyl *cis*-*trans* isomerase	Secondary metabolites
Bcpck1	Phosphoenolpyruvate carboxykinase	Biosynthesis
Bccpr1	NADPH–cytochrome P450 reductase	
Bcser2	Subtilisin-related protease	
Bcsas1	Rab GTPase	Secretory of virulence
BcactA	Actin and related proteins	Factors
Bcsec31	Vesicle coat complex COPII	

### PPI Network of Crotonylated Proteins in *B. cinerea*

**FIGURE 6:**
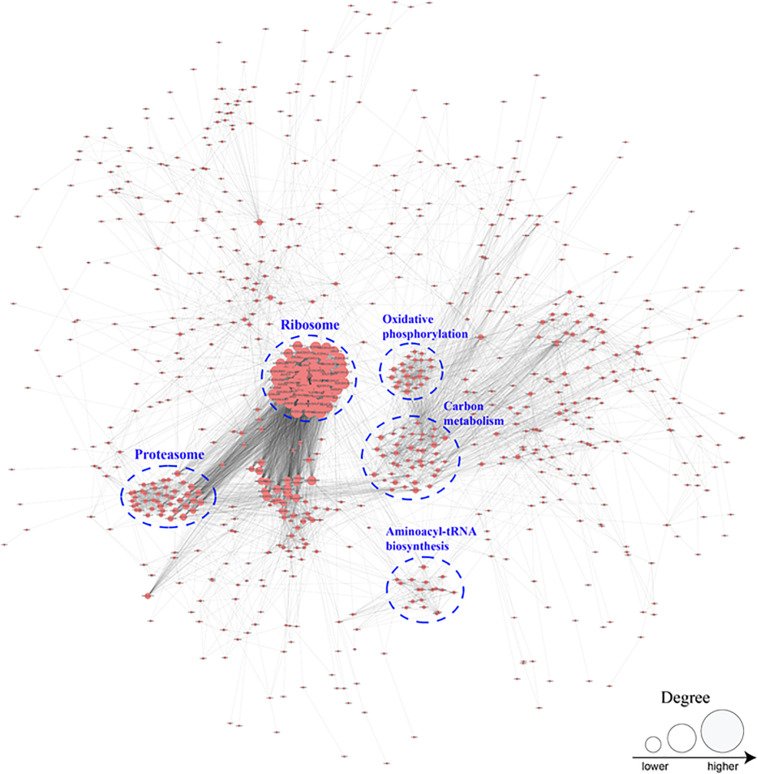
Protein interaction networks of the crotonylation proteins in *B. cinerea*.

To better investigate the cellular processes regulated by crotonylation in *B. cinerea*, a PPI network of all identified crotonylated proteins was assembled using Cytoscape software. As shown in Figure 6 and Supplementary Table S8, 783 crotonylated proteins were mapped to the PPI network, exhibiting an overview of diverse pathways modulated by these proteins in *B. cinerea*. Five greatly interconnected clusters of crotonylated proteins were retrieved, including ribosome, carbon metabolism, proteasome, oxidative phosphorylation, and aminoacyl-tRNA biosynthesis (Figure 6 and Supplementary Table S8). These findings indicate that physiological interactions among these complexes may conduce to their harmonization and cooperation in *B. cinerea*.

### Kcr Levels of *B. cinerea* Under Different Metabolic Conditions

**FIGURE 7:**
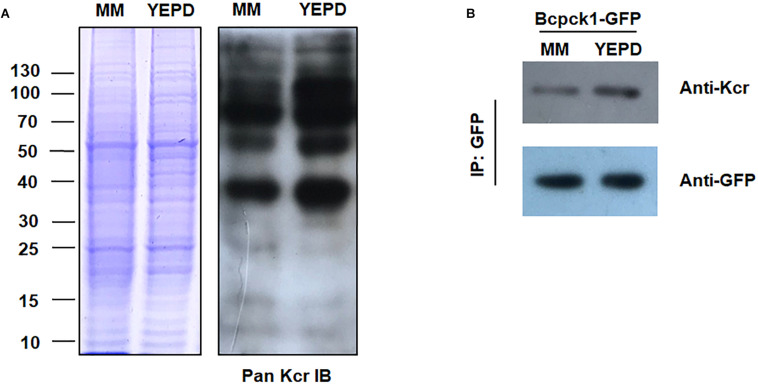
Kcr levels of *B. cinerea* under different physiological conditions. **(A)** Immunoblot analysis of crotonylated proteins with pan anti-Kcr antibody of *B. cinerea* cultivated in liquid YEPD or MM. The loading control by Coomassie blue staining was used to ensure that equal amounts of protein were loaded in each lane. **(B)** Protein extracts containing Bcpck1-GFP were immunoprecipitated (IP) using anti-GFP beads. The isolated proteins were resolved by SDS-PAGE. Anti-GFP and anti-Kcr antibodies were used to detect Bcpck1-GFP and its crotonylated isoform, respectively.

To test whether the crotonylome differs under different physiological conditions, we carried out immunoblotting of the crotonylated proteins of *B. cinerea* cultivated in YEPD or MM (minimal medium), representing rich or limited nutrient condition. As shown in Figure 7A, the whole protein Kcr levels were increased in YEPD than in MM. Moreover, the Kcr level of Bcpck1, a critical enzyme in the nutrient-starvation adaptation before host invasion (Liu J.K. et al., 2018), was also elevated in YEPD (Figure 7B). Thus the above results suggested that Kcr may participate in nutrient metabolic regulation and it will be interesting to characterize the function of crotonylated substrates under different metabolic conditions.

## Discussion

Histone crotonylation is a newly discovered PTM which plays critically important roles for gene transcription in mammalian cells (Tan et al., 2011; Wei et al., 2017a). Other than histones, recent findings on global profiling of crotonylated proteins in various species have expanded the repertoire of modified proteins (Sun et al., 2017, 2019; Wei et al., 2017b; Wu et al., 2017; Xu et al., 2017; Liu J.K. et al., 2018; Liu K. et al., 2018; Liu S. et al., 2018; Yang et al., 2018), which exhibited widely distributed cellular localization and diversified biological function. However, study of Kcr is surprisingly lacking and has not yet been studied in plant fungi. In this study, we performed a global Kcr analysis in *B. cinerea* to bridge this knowledge gap in fungal pathogens.

A total of 1041 crotonylated proteins with 3967 Kcr sites were identified, which exceeds the Kcr levels of most reported organisms or cell lines including *Danio rerio*, *Oryza sativa*, *Nicotiana tabacum*, *Human Hela cells*, *Human peripheral blood*, *Human H1299 cells*, and less than *Human A549 cells* and *Carica papaya*. These data indicate that Kcr is a widespread PTM among organisms. Further functional annotation and enrichment analysis demonstrated that the crotonylated proteins were widely distributed and involved in diverse cellular processes. The identification of specific lysine motifs indicates substrate preference of Kcr in *B. cinerea*. Furthermore, PPI network analysis showed that a mass of protein interactions are regulated by crotonylation. These data represents the first proteome-wide view of the crotonylome in *B. cinerea* and fungal pathogens.

Recently, proteomic studies into crotonylation have indicated that abundant proteins are crotonylated in various organisms, suggesting that crotonylation is a common mechanism of metabolic regulation. However, it remains unclear whether such biochemical changes play crucial roles in physiological processes. In this study, we found that the crotonylome of *B. cinerea* differs under different nutrient conditions (Figure 7). Moreover, the crotonylation of Bcpck1, a PEP-carboxykinase which is important in gluconeogenesis, changed significantly under different metabolic conditions. Future studies are needed to uncover the effects of crotonylation on regulating protein functions and to interpret the underlying mechanisms behind protein crotonylation’s ability to modulate diverse physiological processes.

In our study, 85 ribosomal proteins were crotonylated, accounting for 8% of the total crotonylated proteins, suggesting that Kcr of ribosomal proteins may have effect on protein translational control and ribosome assembly. Afterward, 26 crotonylated proteins were reported to be associated with pathogenicity in *B. cinerea*, participating in various pathogenesis processes (Table 1 and Supplementary Table S7). The infection process of *B. cinerea* is regulated by a complicated signal transduction network, such as Mitogen-activated protein kinases (MAPKs), the cAMP-dependent signal cascade, and small GTPases. In our study, several components involved in these signaling pathways were identified to be crotonylated including MAPKs bmp1 (K298), bmp3 (K72), cAMP-dependent protein kinase Bcpka1 (K205), G-protein alpha subunit Bcg1 (K51,141), and Ras-related small GTPase Bcras1 (K153). Mutants in genes encoding MAPK bmp1 and bmp3 showed defects in germination, vegetative growth, and pathogenicity (Zheng et al., 2000; Rui and Hahn, 2007). Bcpka1 and Bcg1 play important roles in growth and virulence in *B. cinerea*, regulating invasive process of plant tissue via a cAMP-independent pathway (Schumacher et al., 2008a,b). Bcras1, which influences both MAPK and cAMP-dependent signal transduction, is necessary for development and virulence in *B. cinerea* (Minz Dub et al., 2013). These data indicates that Kcr might play a role in signal transduction and thus modulate multiple steps of fungal growth and pathogenicity.

The rapid production of reactive oxygen species (ROS) and maintaining a balanced redox homeostasis are crucial for colonization process of necrotrophic pathogen *B. cinerea*. Cu-Zn superoxide dismutase Bcsod1, an important virulence factor through stimulating ROS generation in *B. cinerea* (López-Cruz et al., 2017), was found to be crotonylated at K89, K106, and K129 sites. Thioredoxin and glutathione are two major cellular redox systems to keep redox homoeostasis during stage of infection for *B. cinerea*. Thioredoxins Bctrx1 (K74, K114), and Bctrx2 (K57), thioredoxin reductase Bctrr1 (K331), and glutathione reductase Bcglr1 (K72, K275, K379) were found to be crotonylated in this study. Knock out mutants of these enzymes showed retarded growth and impaired virulence (Viefhues et al., 2014). In addition, protein disulfide-isomerase Bcpdi1, a new interaction partner of NADPH oxidase complexes which is essential for the conservation of the redox homeostasis in *B. cinerea* (Marschall and Tudzynski, 2017), was identified to be crotonylated at multiple sites in our study.

D-galacturonic acid, one of the major components of plant cell wall, is an important carbon source for filamentous fungi. The mutants in each step of the catabolic pathway of converting D-galacturonic acid of plant to pyruvate and l-glyceraldehyde showed reduced virulence (Zhang et al., 2011). In our study, a group of D-galacturonate catabolic enzymes were identified to be crotonylated including galacturonate reductase Bcgar1 (K276, K286), galactonate dehydratase Bclgd1 (K173, K186, K410), and 2-keto-3-deoxy-l-galactonate aldolase Bclga1 (K247, K307). In addition, endopolygalacturonase Bcpg1, which function as a cell-wall-degrading enzyme and is required for full virulence of *B. cinerea* (ten Have et al., 1998), was found to be crotonylated at multiple sites in this study.

To colonize the host tissue, *B. cinerea* usually secretes a battery of virulence factors to the extracellular environment. The Rab GTPase Bcsas1, reported to play a critical role in the secretion of polygalacturonases, polysaccharide hydrolases, xylanases and proteases (Zhang et al., 2014), was identified to be crotonylated at K172. Deletion of gene encoding vesicle coat complex protein Bcsec31 resulted in a reduction in the protein secretion and virulence of *B. cinerea* (Zhang et al., 2016). This protein was found to be crotonylated at K444 and K571 sites in our study. In addition, actin protein BcactA was found crotonylated at multiple sites, which was recently discovered to influence the secretome of *B. cinerea* and is crucial for extracellular virulence factors regulation (Li et al., 2020).

The secondary metabolites play critical roles in fungal survival and host killing. In our study, several key enzymes responsible for the committed biosynthetic steps were found to be crotonylated. DHN melanin has different effects during the life cycles of the fungus, particularly, contributing to the invading process of the penetration structures and the longevity of the reproduction structures (Zhang et al., 2015; Schumacher, 2016). In this study, three key enzymes of melanogenesis pathway, tetrahydroxynaphthalene reductases Bcbrn1, Bcbrn2, and scytalone dehydratase Bcscd1, were found to be crotonylated at multiple sites. Besides, the phosphoenolpyruvate carboxykinase Bcpck1, which was identified to be crotonylated at multiple sites in our study, plays an important role in gluconeogenesis and is critical in the nutrient-starvation adaptation before host invasion (Liu J.K. et al., 2018). Furthermore, Bccpr1, which function as a cytochrome P450 oxidoreductase regulating the fungal ABA production (Siewers et al., 2004), was identified to be a crotonylated protein with multiple modified sites. Further studies are needed to explore the functions of crotonylation of these substrate proteins to deeply understand mechanisms of *B. cinerea* pathogenesis at protein level.

## Conclusion

We have conducted the first crotonyl-proteome of *B. cinerea*, an important plant fungal pathogen, showing abundant Kcr among a large number of proteins. The lysine crotonylome in this study provides a good resource for in-depth exploration of the functions of Kcr in the growth, development and pathogenicity of *B. cinerea* and other filamentous fungi. Investigating the functions of the crotonylation of the target proteins may help to design drugs with high specificity and low toxicity to prevent and control gray mold disease.

## Data Availability Statement

The mass spectrometry proteomics data have been deposited to the ProteomeXchange Consortium via the PRIDE partner repository with the dataset identifier PXD019358.

## Author Contributions

NZ, WL, and ML generated the hypothesis and planned the experiments. NZ and ZY performed the experiments. NZ and WL wrote the manuscript. All other authors provided comments on the manuscript.

## Conflict of Interest

The authors declare that the research was conducted in the absence of any commercial or financial relationships that could be construed as a potential conflict of interest.

## Supplementary Material

Supplementary Figure 1 **(A)** Systematic analysis of Kcr in *B. cinerea*. **(B)** Mass error distribution of the Kcr peptides. **(C)** Distribution of Kcr peptides based on their length.

Supplementary Figure 2 Overlaps of three individual MS/MS spectra of the crotonylated peptides.

Supplementary Figure 3 MS/MS spectra of three crotonylated peptides: A0A384J6Z0 (Pyruvate kinase), A0A384K3A6 (Bcrpl5), and A0A384K3C6 (Ribosomal protein L15).

Supplementary Figure 4 Number of crotonylation sites per protein in *B. cinerea*.

Supplementary Figure 5 Enrichment analyses of protein domain **(A)** and KEGG pathway **(B)**.

Supplementary Table 1 The identified Kcr sites in *B. cinerea*.

Supplementary Table 2 Conservation analysis of Kcr proteins.

Supplementary Table 3 Protein GO terms distribution.

Supplementary Table 4 GO enrichment based on molecular function, cellular component, and biological process.

Supplementary Table 5 Enrichment analysis of KEGG protein pathway.

Supplementary Table 6 Enrichment analysis of protein domain.

Supplementary Table 7 Crotonylated proteins associated with pathogenicity in *B. cinerea.*

Supplementary Table 8 Protein interaction network of the crotonylated proteins.

## References

[B1] Brandhoff B. Simon A. Dornieden A. Schumacher J. (2017). Regulation of conidiation in *Botrytis cinerea* involves the light-responsive transcriptional regulators BcLTF3 and BcREG1. *Curr. Genet.* 63 931–949. 10.1007/s00294-017-0692-9 28382431

[B2] Chen Y. Sprung R. Tang Y. Ball H. Sangras B. Kim S. C. (2007). Lysine propionylation and butyrylation are novel post-translational modifications in histones. *Mol. Cell Proteomics* 6 812–819. 10.1074/mcp.M700021-MCP200 17267393 PMC2911958

[B3] Chou M. F. Schwartz D. (2011). Biological sequence motif discovery using motif-x. *Curr. Protoc. Bioinformatics* Chapter 13:Unit1315–24. 10.1002/0471250953.bi1315s35 21901740

[B4] Cox J. Mann M. (2008). MaxQuant enables high peptide identification rates, individualized p.p.b.-range mass accuracies and proteome-wide protein quantification. *Nat. Biotechnol.* 26 1367–1372. 10.1038/nbt.1511 19029910

[B5] Cox J. Matic I. Hilger M. Nagaraj N. Selbach M. Olsen J. V. (2009). A practical guide to the MaxQuant computational platform for SILAC-based quantitative proteomics. *Nat. Protoc.* 4 698–705. 10.1038/nprot.2009.36 19373234

[B6] Dai L. Peng C. Montellier E. Lu Z. Chen Y. Ishii H. (2014). Lysine 2-hydroxyisobutyrylation is a widely distributed active histone mark. *Nat. Chem. Biol.* 10 365–370. 10.1038/nchembio.1497 24681537

[B7] Ebrecht A. C. Van Der Bergh N. Harrison S. T. L. Smit M. S. Sewell B. T. Opperman D. J. (2019). Biochemical and structural insights into the cytochrome P450 reductase from Candida tropicalis. *Sci. Rep.* 9:20088. 10.1038/s41598-019-56516-6 31882753 PMC6934812

[B8] Gronover C. S. Kasulke D. Tudzynski P. Tudzynski B. (2001). The role of G protein alpha subunits in the infection process of the gray mold fungus *Botrytis cinerea*. *Mol. Plant Microbe Interact.* 14 1293–1302. 10.1094/mpmi.2001.14.11.1293 11763127

[B9] Hirschey M. D. Zhao Y. (2015). Metabolic regulation by lysine malonylation, succinylation, and glutarylation. *Mol. Cell Proteomics* 14 2308–2315. 10.1074/mcp.R114.046664 25717114 PMC4563717

[B10] Horton P. Park K. J. Obayashi T. Fujita N. Harada H. Adams-Collier C. J. (2007). WoLF PSORT: protein localization predictor. *Nucleic Acids Res.* 35 W585–W587. 10.1093/nar/gkm259 17517783 PMC1933216

[B11] Huang H. Wang D. L. Zhao Y. (2018). Quantitative crotonylome analysis expands the roles of p300 in the regulation of lysine crotonylation pathway. *Proteomics* 18:e1700230. 10.1002/pmic.201700230 29932303 PMC6420807

[B12] Kanehisa M. Goto S. Kawashima S. Okuno Y. Hattori M. (2004). The KEGG resource for deciphering the genome. *Nucleic Acids Res.* 32 D277–D280. 10.1093/nar/gkh063 14681412 PMC308797

[B13] Khoury G. A. Baliban R. C. Floudas C. A. (2011). Proteome-wide post-translational modification statistics: frequency analysis and curation of the swiss-prot database. *Sci. Rep.* 1:90. 10.1038/srep00090 22034591 PMC3201773

[B14] Kwon O. K. Kim S. J. Lee S. (2018). First profiling of Kcr of myofilament proteins and ribosomal proteins in zebrafish embryos. *Sci. Rep.* 8:3652. 10.1038/s41598-018-22069-3 29483630 PMC5827021

[B15] Leroch M. Mernke D. Koppenhoefer D. Schneider P. Mosbach A. Doehlemann G. (2011). Living colors in the gray mold pathogen *Botrytis cinerea*: codon-optimized genes encoding green fluorescent protein and mCherry, which exhibit bright fluorescence. *Appl. Environ. Microbiol.* 77 2887–2897. 10.1128/aem.02644-10 21378036 PMC3126427

[B16] Li D. Lv B. Tan L. Yang Q. Liang W. (2016). Acetylome analysis reveals the involvement of lysine acetylation in diverse biological processes in Phytophthora sojae. *Sci. Rep.* 6:29897. 10.1038/srep29897 27412925 PMC4944153

[B17] Li H. Zhang Z. Qin G. He C. Li B. Tian S. (2020). actin is required for cellular development and virulence of *Botrytis cinerea* via the mediation of secretory proteins. *mSystems* 5:e00732-19. 10.1128/mSystems.00732-19 32098836 PMC7043344

[B18] Liu J. K. Chang H. W. Liu Y. Qin Y. H. Ding Y. H. Wang L. (2018). The key gluconeogenic gene PCK1 is crucial for virulence of *Botrytis cinerea* via initiating its conidial germination and host penetration. *Environ. Microbiol.* 20 1794–1814. 10.1111/1462-2920.14112 29614212

[B19] Liu K. Yuan C. Li H. Chen K. Lu L. Shen C. (2018). A qualitative proteome-wide Kcr profiling of papaya (*Carica papaya* L.). *Sci. Rep.* 8:8230. 10.1038/s41598-018-26676-y 29844531 PMC5974297

[B20] Liu S. Xue C. Fang Y. Chen G. Peng X. Zhou Y. (2018). Global involvement of lysine crotonylation in protein modification and transcription regulation in rice. *Mol. Cell Proteomics* 17 1922–1936. 10.1074/mcp.RA118.000640 30021883 PMC6166680

[B21] Liu L. Wang G. Song L. Lv B. Liang W. (2016). Acetylome analysis reveals the involvement of lysine acetylation in biosynthesis of antibiotics in Bacillus amyloliquefaciens. *Sci. Rep.* 6:20108. 10.1038/srep20108 26822828 PMC4731788

[B22] López-Cruz J. Óscar C. S. Emma F. C. Pilar G. A. Carmen G. B. (2017). Absence of Cu-Zn superoxide dismutase BCSOD1 reduces *Botrytis cinerea* virulence in *Arabidopsis* and tomato plants, revealing interplay among reactive oxygen species, callose and signalling pathways. *Mol. Plant Pathol.* 18 16–31. 10.1111/mpp.12370 26780422 PMC6638242

[B23] Lv B. Yang Q. Li D. Liang W. Song L. (2016). Proteome-wide analysis of lysine acetylation in the plant pathogen *Botrytis cinerea*. *Sci. Rep.* 6:29313. 10.1038/srep29313 27381557 PMC4933888

[B24] Marschall R. Tudzynski P. (2017). The protein disulfide isomerase of *Botrytis cinerea*: an ER protein involved in protein folding and redox homeostasis influences NADPH oxidase signaling processes. *Front. Microbiol.* 8:960. 10.3389/fmicb.2017.00960 28611757 PMC5447010

[B25] Minz Dub A. Kokkelink L. Tudzynski B. Tudzynski P. Sharon A. (2013). Involvement of *Botrytis cinerea* small GTPases BcRAS1 and BcRAC in differentiation, virulence, and the cell cycle. *Eukaryot Cell* 12 1609–1618. 10.1128/ec.00160-13 24096906 PMC3889569

[B26] Porquier A. Moraga J. Morgant G. Dalmais B. Simon A. Sghyer H. (2019). Botcinic acid biosynthesis in *Botrytis cinerea* relies on a subtelomeric gene cluster surrounded by relics of transposons and is regulated by the Zn(2)Cys(6) transcription factor BcBoa13. *Curr. Genet.* 65 965–980. 10.1007/s00294-019-00952-4 30848345

[B27] Rui O. Hahn M. (2007). The Slt2-type MAP kinase Bmp3 of *Botrytis cinerea* is required for normal saprotrophic growth, conidiation, plant surface sensing and host tissue colonization. *Mol. Plant Pathol.* 8 173–184. 10.1111/j.1364-3703.2007.00383.x 20507489

[B28] Schumacher J. (2016). DHN melanin biosynthesis in the plant pathogenic fungus *Botrytis cinerea* is based on two developmentally regulated key enzyme (PKS)-encoding genes. *Mol. Microbiol.* 99 729–748. 10.1111/mmi.13262 26514268

[B29] Schumacher J. Kokkelink L. Huesmann C. Jimenez-Teja D. Collado I. G. Barakat R. (2008a). The cAMP-dependent signaling pathway and its role in conidial germination, growth, and virulence of the gray mold *Botrytis cinerea*. *Mol. Plant Microbe Interact.* 21 1443–1459. 10.1094/mpmi-21-11-1443 18842094

[B30] Schumacher J. Viaud M. Simon A. Tudzynski B. (2008b). The Galpha subunit BCG1, the phospholipase C (BcPLC1) and the calcineurin phosphatase co-ordinately regulate gene expression in the grey mould fungus *Botrytis cinerea*. *Mol. Microbiol.* 67 1027–1050. 10.1111/j.1365-2958.2008.06105.x 18208491

[B31] Shannon P. Markiel A. Ozier O. Baliga N. S. Wang J. T. Ramage D. (2003). Cytoscape: a software environment for integrated models of biomolecular interaction networks. *Genome Res.* 13 2498–2504. 10.1101/gr.1239303 14597658 PMC403769

[B32] Siewers V. Smedsgaard J. Tudzynski P. (2004). The P450 monooxygenase BcABA1 is essential for abscisic acid biosynthesis in *Botrytis cinerea*. *Appl. Environ. Microbiol.* 70 3868–3876. 10.1128/aem.70.7.3868-3876.2004 15240257 PMC444755

[B33] Sun H. Liu X. Li F. Li W. Zhang J. Xia Z. (2017). First comprehensive proteome analysis of Kcr in seedling leaves of Nicotiana tabacum. *Sci. Rep.* 7:3013. 10.1038/s41598-017-03369-6 28592803 PMC5462846

[B34] Sun J. Qiu C. Qian W. Wang Y. Sun L. Li Y. (2019). Ammonium triggered the response mechanism of lysine crotonylome in tea plants. *BMC Genomics* 20:340. 10.1186/s12864-019-5716-z 31060518 PMC6501322

[B35] Szklarczyk D. Franceschini A. Wyder S. Forslund K. Heller D. Huerta-Cepas J. (2015). STRING v10: protein-protein interaction networks, integrated over the tree of life. *Nucleic Acids Res.* 43 D447–D452. 10.1093/nar/gku1003 25352553 PMC4383874

[B36] Tan M. Luo H. Lee S. Jin F. Yang J. S. Montellier E. (2011). Identification of 67 histone marks and histone Kcr as a new type of histone modification. *Cell* 146 1016–1028. 10.1016/j.cell.2011.08.008 21925322 PMC3176443

[B37] ten Have A. Mulder W. Visser J. Van Kan J. A. (1998). The endopolygalacturonase gene Bcpg1 is required for full virulence of *Botrytis cinerea*. *Mol. Plant Microbe Interact.* 11 1009–1016. 10.1094/mpmi.1998.11.10.1009 9768518

[B38] Viefhues A. Heller J. Temme N. Tudzynski P. (2014). Redox systems in *Botrytis cinerea*: impact on development and virulence. *Mol. Plant Microbe Interact.* 27 858–874. 10.1094/mpmi-01-14-0012-r 24983673

[B39] Wang M. Weiberg A. Dellota E. Jr. Yamane D. Jin H. (2017). Botrytis small RNA Bc-siR37 suppresses plant defense genes by cross-kingdom RNAi. *RNA Biol.* 14 421–428. 10.1080/15476286.2017.1291112 28267415 PMC5411126

[B40] Wang Y. Zhou J. Zhong J. Luo D. Li Z. Yang J. (2018). Cys(2)His(2) zinc finger transcription factor BcabaR1 positively regulates abscisic acid production in *Botrytis cinerea*. *Appl. Environ. Microbiol.* 84:AEM.00920-18. 10.1128/aem.00920-18 29959241 PMC6102986

[B41] Wei W. Liu X. Chen J. Gao S. Lu L. Zhang H. (2017a). Class I histone deacetylases are major histone decrotonylases: evidence for critical and broad function of histone crotonylation in transcription. *Cell Res.* 27 898–915. 10.1038/cr.2017.68 28497810 PMC5518989

[B42] Wei W. Mao A. Tang B. Zeng Q. Gao S. Liu X. (2017b). Large-scale identification of protein crotonylation reveals its role in multiple cellular functions. *J. Proteome Res.* 16 1743–1752. 10.1021/acs.jproteome.7b00012 28234478

[B43] Weiberg A. Wang M. Lin F. M. Zhao H. Zhang Z. Kaloshian I. (2013). Fungal small RNAs suppress plant immunity by hijacking host RNA interference pathways. *Science* 342 118–123. 10.1126/science.1239705 24092744 PMC4096153

[B44] Williamson B. Tudzynski B. Tudzynski P. Van Kan J. A. (2007). *Botrytis cinerea*: the cause of grey mould disease. *Mol. Plant Pathol.* 8 561–580. 10.1111/j.1364-3703.2007.00417.x 20507522

[B45] Wu Q. Li W. Wang C. Fan P. Cao L. Yang J. (2017). Ultradeep lysine crotonylome reveals the crotonylation enhancement on both histones and nonhistone proteins by SAHA treatment. *J. Proteome Res.* 16 3664–3671. 10.1021/acs.jproteome.7b00380 28882038

[B46] Xu W. Wan J. Zhan J. Li X. He H. Shi Z. (2017). Global profiling of crotonylation on non-histone proteins. *Cell Res.* 27 946–949. 10.1038/cr.2017.60 28429772 PMC5518986

[B47] Yang Q. Li Y. Apaliya M. T. Zheng X. Serwah B. N. A. Zhang X. (2018). The response of rhodotorula mucilaginosa to patulin based on lysine crotonylation. *Front. Microbiol.* 9:2025. 10.3389/fmicb.2018.02025 30233516 PMC6129574

[B48] Yu J. Zhou C. Z. (2007). Crystal structure of glutathione reductase Glr1 from the yeast *Saccharomyces cerevisiae*. *Proteins* 68 972–979. 10.1002/prot.21354 17554778

[B49] Zhang C. He Y. Zhu P. Chen L. Wang Y. Ni B. (2015). Loss of bcbrn1 and bcpks13 in *Botrytis cinerea* not only blocks melanization but also increases vegetative growth and virulence. *Mol. Plant Microbe Interact.* 28 1091–1101. 10.1094/mpmi-04-15-0085-r 26035129

[B50] Zhang D. Tang Z. Huang H. Zhou G. Cui C. Weng Y. (2019). Metabolic regulation of gene expression by histone lactylation. *Nature* 574 575–580. 10.1038/s41586-019-1678-1 31645732 PMC6818755

[B51] Zhang L. Thiewes H. Van Kan J. A. (2011). The D-galacturonic acid catabolic pathway in *Botrytis cinerea*. *Fungal Genet. Biol.* 48 990–997. 10.1016/j.fgb.2011.06.002 21683149

[B52] Zhang Z. Li H. Qin G. He C. Li B. Tian S. (2016). The MADS-Box transcription factor Bcmads1 is required for growth, sclerotia production and pathogenicity of *Botrytis cinerea*. *Sci. Rep.* 6:33901. 10.1038/srep33901 27658442 PMC5034256

[B53] Zhang Z. Qin G. Li B. Tian S. (2014). Knocking out Bcsas1 in *Botrytis cinerea* impacts growth, development, and secretion of extracellular proteins, which decreases virulence. *Mol. Plant Microbe Interact.* 27 590–600. 10.1094/mpmi-10-13-0314-r 24520899

[B54] Zheng L. Campbell M. Murphy J. Lam S. Xu J. R. (2000). The BMP1 gene is essential for pathogenicity in the gray mold fungus *Botrytis cinerea*. *Mol. Plant Microbe Interact.* 13 724–732. 10.1094/mpmi.2000.13.7.724 10875333

[B55] Zhou S. Yang Q. Yin C. Liu L. Liang W. (2016). Systematic analysis of the lysine acetylome in *Fusarium graminearum*. *BMC Genomics* 17:1019. 10.1186/s12864-016-3361-3 27964708 PMC5153868

