# Supplementary figures and images for: Global Proteomic Analysis of Lysine Crotonylation in the Plant Pathogen Botrytis cinerea
Source: Front Microbiol. 2020 Oct 23;11:564350. doi: 10.3389/fmicb.2020.564350 (PMC7644960; doi:10.3389/fmicb.2020.564350)

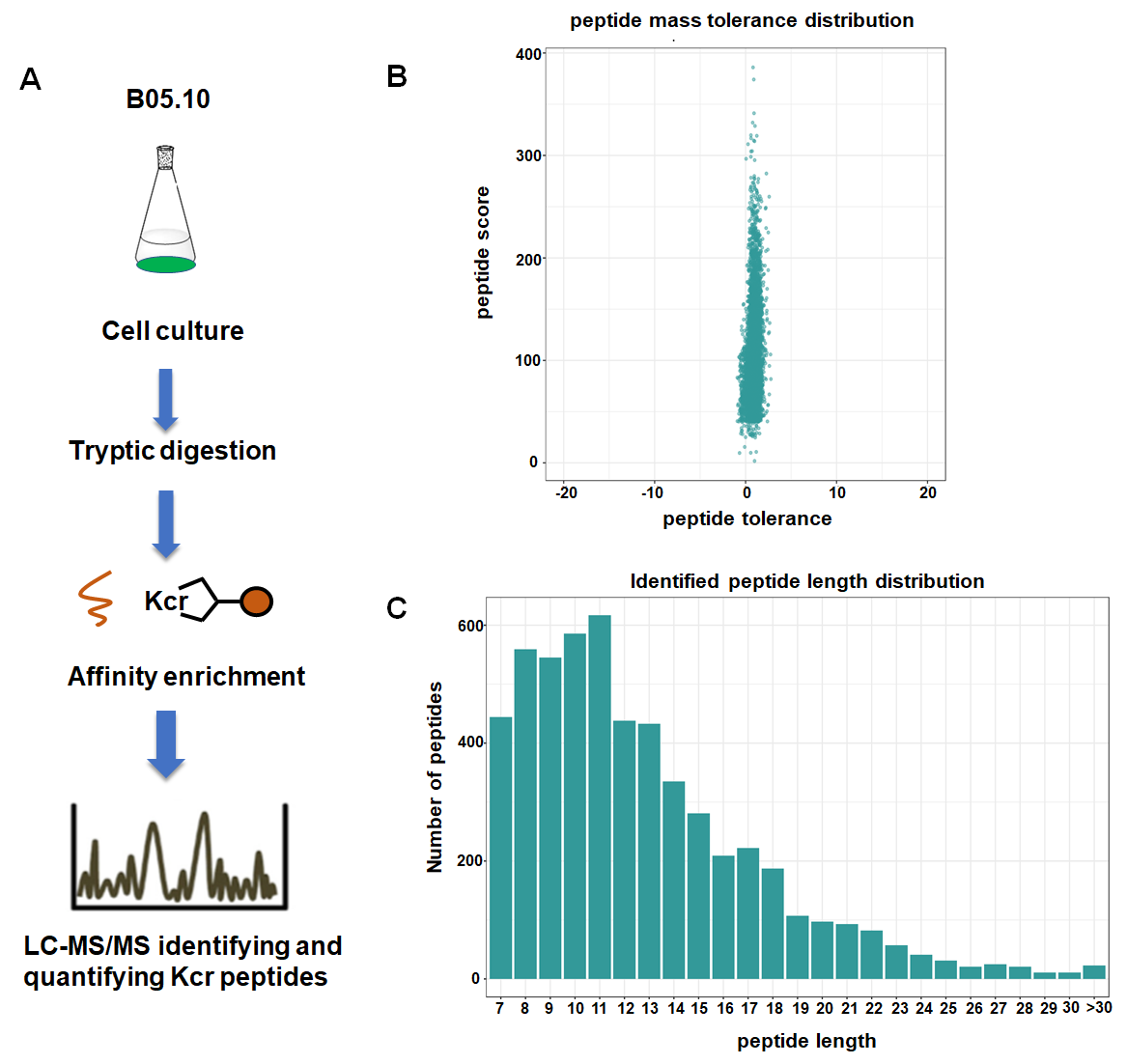

Supplement: Supplementary Figure 1 — (A) Systematic analysis of Kcr in B. cinerea. (B) Mass error distribution of the Kcr peptides. (C) Distribution of Kcr peptides based on their length. [file Image_1.TIF]

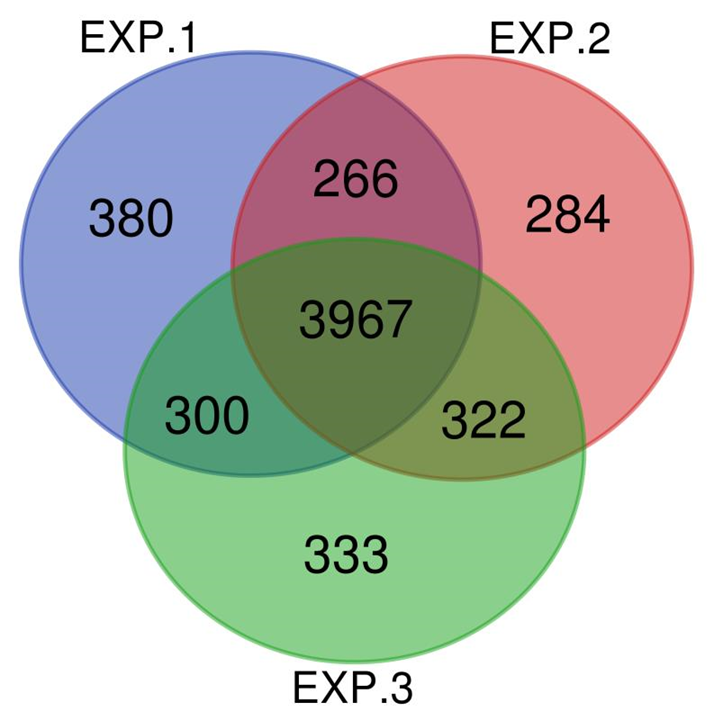

Supplement: Supplementary Figure 2 — Overlaps of three individual MS/MS spectra of the crotonylated peptides. [file Image_2.TIF]

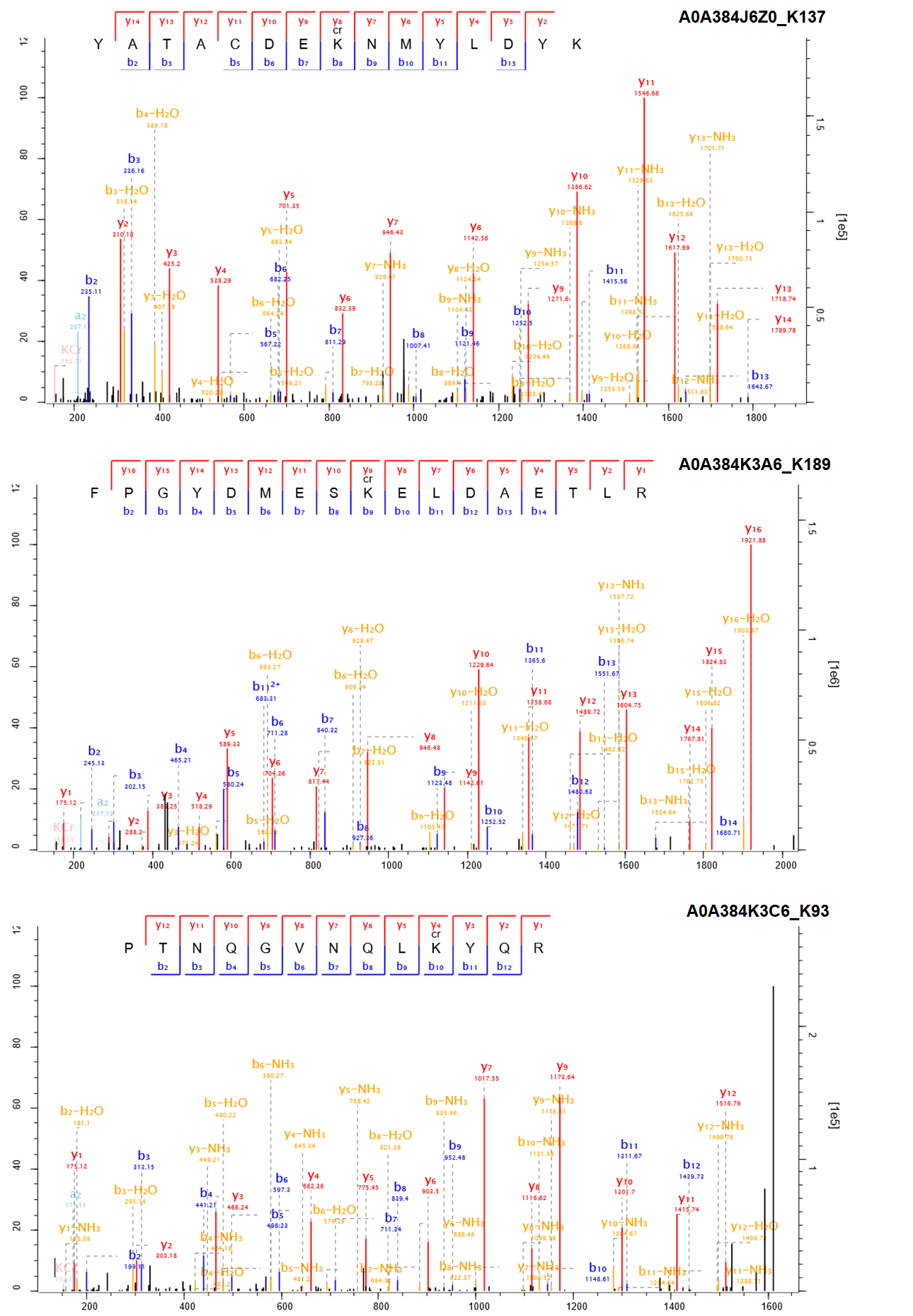

Supplement: Supplementary Figure 3 — MS/MS spectra of three crotonylated peptides: A0A384J6Z0 (Pyruvate kinase), A0A384K3A6 (Bcrpl5), and A0A384K3C6 (Ribosomal protein L15). [file Image_3.TIF]

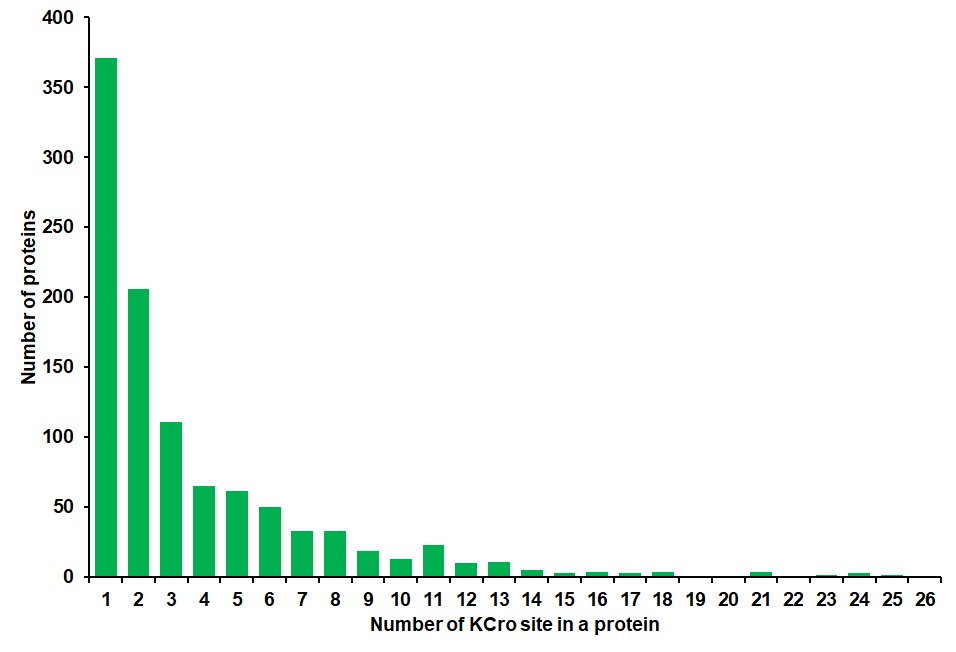

Supplement: Supplementary Figure 4 — Number of crotonylation sites per protein in B. cinerea. [file Image_4.TIF]

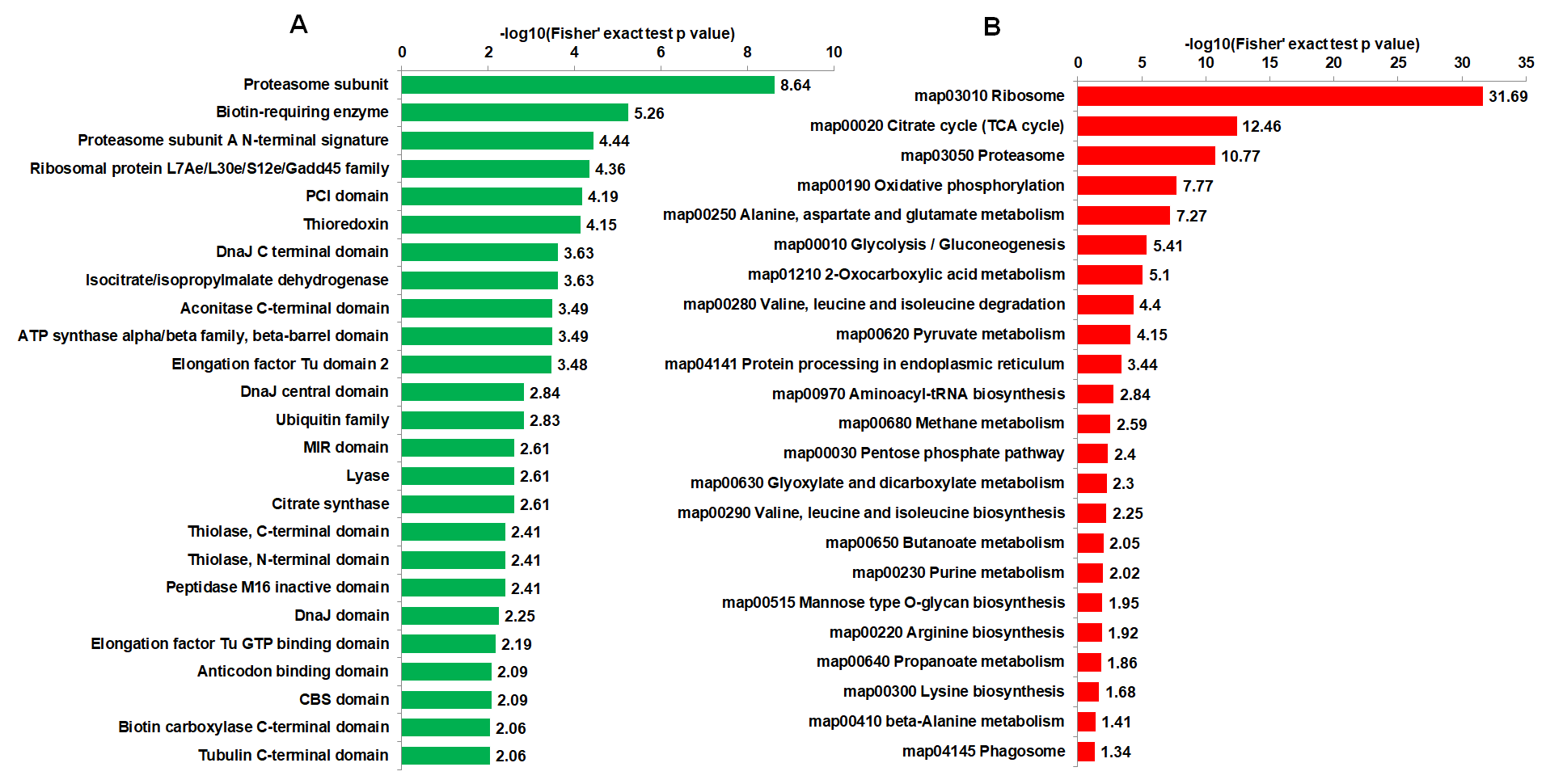

Supplement: Supplementary Figure 5 — Enrichment analyses of protein domain (A) and KEGG pathway (B). [file Image_5.TIF]
